# Light Chain Amyloid Fibrils Cause Metabolic Dysfunction in Human Cardiomyocytes

**DOI:** 10.1371/journal.pone.0137716

**Published:** 2015-09-22

**Authors:** Helen P. McWilliams-Koeppen, James S. Foster, Nicole Hackenbrack, Marina Ramirez-Alvarado, Dallas Donohoe, Angela Williams, Sallie Macy, Craig Wooliver, Dale Wortham, Jennifer Morrell-Falvey, Carmen M. Foster, Stephen J. Kennel, Jonathan S. Wall

**Affiliations:** 1 Department of Medicine, University of Tennessee Graduate School of Medicine, Knoxville, TN United States of America; 2 Department of Radiology, University of Tennessee Graduate School of Medicine, Knoxville, TN United States of America; 3 Department of Biochemistry/Mol. Biol. and Immunology, Mayo Clinic, Rochester, MN, United States of America; 4 Department of Nutrition, University of Tennessee Knoxville, TN, United States of America; 5 Biosciences Division, Oak Ridge National Laboratory, Oak Ridge, TN, United States of America; Kermanshah University of Medical Sciences, ISLAMIC REPUBLIC OF IRAN

## Abstract

Light chain (AL) amyloidosis is the most common form of systemic amyloid disease, and cardiomyopathy is a dire consequence, resulting in an extremely poor prognosis. AL is characterized by the production of monoclonal free light chains that deposit as amyloid fibrils principally in the heart, liver, and kidneys causing organ dysfunction. We have studied the effects of amyloid fibrils, produced from recombinant λ6 light chain variable domains, on metabolic activity of human cardiomyocytes. The data indicate that fibrils at 0.1 μM, but not monomer, significantly decrease the enzymatic activity of cellular NAD(P)H-dependent oxidoreductase, without causing significant cell death. The presence of amyloid fibrils did not affect ATP levels; however, oxygen consumption was increased and reactive oxygen species were detected. Confocal fluorescence microscopy showed that fibrils bound to and remained at the cell surface with little fibril internalization. These data indicate that AL amyloid fibrils severely impair cardiomyocyte metabolism in a dose dependent manner. These data suggest that effective therapeutic intervention for these patients should include methods for removing potentially toxic amyloid fibrils.

## Introduction

Immunoglobulin light chain amyloidosis (AL) is a disease wherein plasma cell-derived monoclonal light chains (LC) are secreted into the circulation and self-aggregate into amyloid fibrils that deposit in peripheral organs causing dysfunction and often death [1–4]. Amyloidogenic LC proteins are characterized by a metastable folding state that arises due to destabilizing mutations or post-translational modification, resulting in an increased propensity for misfolding and amyloid fibril formation [5–8]. The prognoses in patients with AL and treatment stratification are related to the amyloid load and organ distribution [9, 10].

**Fig 1:**
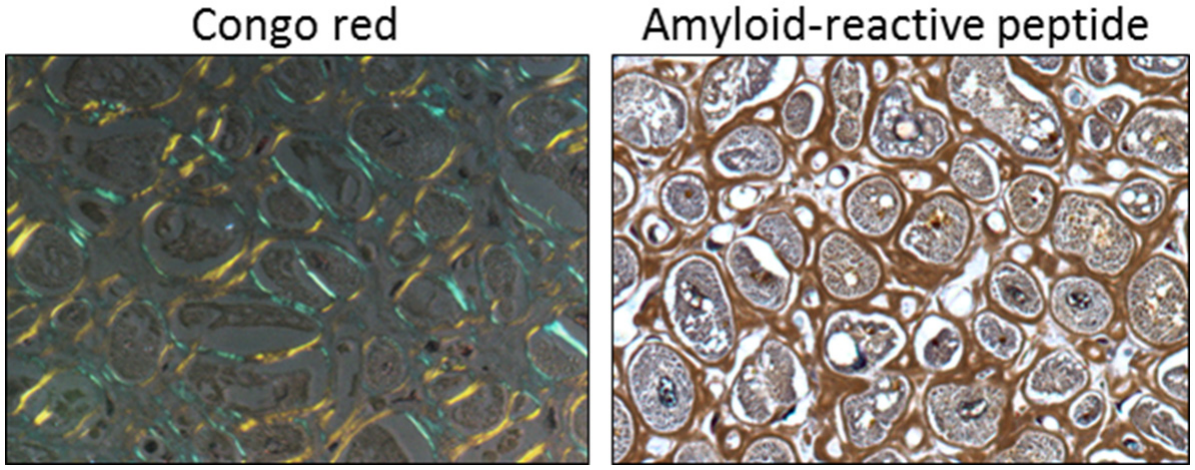
AL amyloid cardiomyopathy. Infiltrative amyloid encompassing human cardiomyocytes, seen as blue-gold birefringent extracellular material when stained with Congo red (left) or brown deposits when stained with a biotinylated amyloid-reactive peptide (right).

AL amyloid cardiomyopathy is associated with the deposition of amyloid, in the atria, ventricles, and coronary vessels and is the most ominous clinical manifestation of this rare, but often fatal disease [10, 11]. Approximately 50% of AL patients present with cardiac involvement and, if left untreated, the median survival is ~ 6 mos [9, 11]. Unlike other forms of restrictive cardiomyopathy, cardiac amyloidosis cannot be treated successfully with standard therapies (e.g., digoxin, β-blockers, or ACE inhibitors) due to excessive toxicity and the potential for profound hypertension in these patients [12]. Restrictive AL amyloid cardiomyopathy is associated with increased serum levels of cardiac troponin and brain natriuretic peptide (BNP) and a characteristic interventricular septal wall thickening due to amyloid infiltration (Fig 1), although there is little evidence of chronic cardiomyocyte apoptosis [12–14]. Cardiac amyloid deposits invariably contribute to loss of ventricular elasticity and impaired relaxation in AL, yet it is worth considering that AL amyloid fibrils, or related components, such as the free LC, or oligomeric forms thereof, also cause cardiomyocyte dysfunction and exacerbate cardiac insufficiency. AL patients generally have high concentrations of circulating monoclonal LC in addition to tissue amyloid deposits.

Cytotoxicity is an unequivocal hallmark of at least two amyloid-related disorders, Alzheimer’s disease and type 2 diabetes, where intracellular and extracellular oligomeric species of the Aβ and islet amyloid polypeptide (IAPP) peptides cause death of neurons and pancreatic islet β-cells, respectively [15–18]. For these two localized amyloidoses, the mature amyloid fibrils themselves are often considered to be less toxic, benign, or even protective with respect to cellular health. Although evidence for cytotoxicity in systemic amyloidosis remains less well characterized, toxic oligomeric species of transthyretin have recently been identified, which presumably underlie the neuropathy seen in patients with transthyretin-associated amyloidosis [19, 20]. There is, however, no definitive evidence of circulating cytotoxic LC oligomers in AL patients.

In support of a LC component contributing to cardiac dysfunction, patients with a hematologic response to high-dose, plasma cell chemotherapy (i.e. a reduction in circulating free LC concentration) exhibited improved cardiac health, as indicated by a decrease in the serum brain natriuretic protein (BNP) concentration [21] as a marker of cardiomyocyte dysfunction [22]. Thus, circulating LC may play a detrimental role in cardiac function although the reduction in circulating light chains might also yield improvement by promoting disaggregation of the amyloid deposits.

Light chain preparations isolated from the urine of patients with cardiac AL, have been shown to cause oxidative stress in primary rat cardiomyocytes in the absence of detectable aggregation or amyloid fibril formation [23, 24]. Furthermore, LC from patients with multiple myeloma, or those lacking severe cardiac involvement did not produce the same effect [23]. Sikkink *et al*., working with HL-1 mouse cardiomyocytes, demonstrated that recombinant LC κ1 variable domain (rVκ1) proteins caused a decrease in MTT reduction and were internalized in mouse cardiomyocytes with no loss of cell viability [25]. Preclinical evidence for toxicity of circulating LC *in vivo* has been presented in cardiac tissue explants [26] and in a zebrafish model [27]. The absence of a robust mammalian model of AL, exhibiting cardiomyopathy, has hampered progress in defining the toxic specie(s) or the mechanism of action. *In vitro* studies related to the direct effects of AL fibrils on cardiomyocyte metabolism have not been reported. Furthermore, no study has compared the effect of soluble proteins with their corresponding amyloid fibrils.

Herein, we have revisited the question of AL amyloid-related cardiotoxicity by examining the effect of amyloid fibrils, composed of recombinant λ6 LC variable domains (rVλ6), as well as the non-fibrillar form of the proteins, on metabolic functions of cultured human cardiomyocytes. These rVλ6 proteins are excellent substrates for this study as they are among the most fibrillogenic proteins thus far described [6, 7, 28, 29]. Furthermore, these proteins exhibit essentially complete conversion of monomer to the fibrils when agitated under physiological conditions at room temperature [7, 30]. We demonstrate that fibrils bound tightly to the cell surface and that this interaction resulted in a significant decrease in cellular NAD(P)H-dependent oxidoreductase activity as measured by MTT reduction, an induction of reactive oxygen species, and an increase in the oxygen consumption rate. In contrast to the effects seen with fibrillar Vλ6 preparations, the non-fibrillar precursor Vλ6 proteins were relatively benign when added to the cells in culture even at higher concentrations. Additionally, fibril-mediated metabolic dysfunction in cultured human cardiomyocytes did not result in significant cell death.

## Methods and Materials

### Materials

AC10 human ventricular cardiomyocytes (PTA-1501) and DMEM:F12 medium (supplemented with 2.5 mM glutamine, 15 mM HEPES, 0.5mM sodium pyruvate, and 1200 mg/L sodium bicarbonate) were obtained from the ATCC (Manassas, VA). Characterized fetal bovine serum (FBS) and Dulbecco’s phosphate-buffered saline without Ca^2+^ and Mg^2+^ (PBS), and thioflavin T (ThT) were purchased from Thermo Scientific (Waltham, MA). Hank’s balanced salt solution, 100x penicillin-streptomycin solution, L-glutamine, trypsin, and EDTA were purchased from Corning Life Sciences (Tewksbury, MA). 3-(4,5-dimethylthiazol-2-yl)-2,5-diphenyltetrazolium bromide (MTT), dichloro-dihydro-fluorescein diacetate (DCFH-DA), elastin, crystal violet, and anhydrous dimethyl sulfoxide (DMSO) were purchased from Sigma (St. Louis, MO). Cellular ATP quantitation was performed with the CellTiter-Glo Luminescent Cell Viability Assay kit from Promega (Madison, WI) according to the manufacturer’s directions. Hoescht 33342, Alexa Fluor 488 NHS Ester (succinimidyl ester), and Alexa Fluor 647 phalloidin were purchased from Life Technologies (Grand Island, NY). BD Cytofix was from Becton-Dickinson (Franklin Lakes, NJ). 5-chloromethylfluorescein diacetate (CMFDA) was purchased from Genecopoeia (Rockville, MD).

### Cell Culture

The AC10 cell line is one of four clones of primary adult human ventricular cardiomyocytes that were immortalized via SV40 transformation by Davidson *et al*. [31]. These cells have been shown to express cardiomyocyte markers, including α- and β- myosin heavy chain, α-cardiac actin, myofilaments, troponin I, desmin, BNP, and functional gap junction proteins. This human cardiomyocyte model offers a more robust *in vitro* system for translational human medicine than alternative cell lines of murine origin [32].

AC10 cardiomyocytes were seeded at 1 x 10^4^/cm^2^ in T75 culture flasks (Corning Life Sciences, Tewksbury, MA) and propagated in DMEM:F12 containing 12.5% FBS, 50 IU penicillin, 50 μg/mL streptomycin, and 4.5 mM L-glutamine (growth medium), at 37°C and 5% CO_2_, [31]. To subculture, cells were detached with 0.25% trypsin/2.21 mM EDTA for one minute at 37°C, suspended in growth medium, centrifuged at 200 x g, and resuspended in growth medium for cell counting and viability determination. In all assay formats, serum-free DMEM:F12 medium was used during the incubation of cells with rVλ6 fibrils and monomer.

### Preparation of recombinant (r) λ6 variable domain proteins and fibrils

Recombinant Vλ6 proteins Wil, Jto, and point mutant, JtoR68S, were prepared from a periplasmic extract of transformed *E*. *coli*, and purified, as previously described [7, 30]. rVλ6 fibrils were prepared as described [33]. Briefly, lyophilized rVλ6 protein was reconstituted with ultrapure water, adjusted to 1X PBS, and filtered via 0.22-μm pore-sized PVDF syringe filters (EMD Millipore, Billerica, MA). Protein concentration was measured spectrophotometrically at 280 nm (using calculated extinction coefficients (ε_280_) of 12090 and 13370 for rVλ6Wil and rVλ6Jto/rVλ6JtoR68S, respectively) and suspensions diluted to a final concentration of 1 mg/mL. Monomeric rVλ6 preparations were prepared freshly before use or snap frozen in liquid N_2_ and stored at -80°C for no more than one week before being used in cytotoxicity assays. To prepare amyloid fibrils, a 1 mL-volume of monomer suspension was placed in a 15 mL conical polypropylene tube (BD Biosciences, Franklin Lakes, NJ) and shaken at a 45° angle at 225 rpm for 72 h at 37°C [33]. If necessary, samples were taken during the course of fibrillogenesis, snap frozen, and stored at -80°C. The presence of fibrils was documented by measuring the fluorescence emission at 490 nm following the addition of 50 μM ThT [33]. Fibril preparations were aliquoted into single use volumes, and stored at -80°C. For preparation of fluorescently labeled rVλ6Wil monomer, the protein (1 mg/mL) was labeled with Alexa Fluor 488 NHS (N-hydroxy-succinimidyl) ester per the manufacturer’s instructions and dialyzed against PBS. Fluorescent- rV_L_λ6Wil fibrils were prepared as described above using a 10:1 molar ratio mixture of unlabeled monomer with Alexa-Fluor 488-labeled rVλ6Wil.

Fibrillogenesis reactions were also monitored by measuring the amount of free monomer in the reaction supernatant following ultracentrifugation at 435,000 x g by using RP-HPLC with a Zorbax SB-C3 solid phase (Agilent Technologies, Santa Clara, CA) and a 0–51% (v/v) acetonitrile gradient (2% per min) mobile phase.

### Electron microscopy

The rVλ6 fibril ultrastructure was investigated by using electron microscopy. Briefly, rVLλ6 fibrils were allowed to settle onto a thin carbon film on a copper grid, washed, and negatively-stained using 1% uranyl acetate. Digital images of representative fields of protein were acquired using a Zeiss Libra 200MC transmission electron microscope.

### MTT assay

AC10 cardiomyocytes were seeded at 2 x 10^4^/cm^2^ in 24-well tissue culture-treated plates (Corning Life Sciences, Tewksbury, MA) and grown for 24 h in growth medium. At 24 h, growth medium was replaced with serum-free DMEM:F12, followed by the addition in triplicate of rVλ6 fibrils or monomer, or an equivalent volume of PBS as a control. Cells were cultured in the presence of rVλ6 fibrils or monomer for an additional 20 h, after which MTT in PBS, filtered with a 0.22 μm syringe filter, was added to the cells to yield a final concentration of 0.5 mg/mL. After a 4 h incubation with MTT at 37°C, the culture medium was aspirated and the plates air-dried. The formazan precipitate was dissolved in isopropanol (0.4 mL) and the absorbance at 595 nm quantified using a microplate reader (Synergy HT, Biotek, Winooski, VT). The average absorbance of the PBS-treated wells was used to establish a 100% activity value and changes in the treated samples were expressed as a percentage of this value.

### Measurement of cell number and viability

Cell density was determined using a crystal violet staining assay [34, 35]. Briefly, after incubation with 1 μM rVλ6Wil fibrils, the experimental medium was aspirated from the 24-wellplate and cells were fixed for 10 min with 1% glutaraldehyde in PBS. After the removal of glutaraldehyde, the cell monolayer was washed once with deionized water, and the plates were air-dried. The fixed cells were stained with a 0.2% solution of crystal violet in water for 30 min. After staining, the cells were washed three times with deionized water and the cell-associated stain was dissolved in 10% acetic acid (0.4 mL) and quantified by measuring the absorbance at 595 nm (Synergy HT). In parallel, cell viability was assessed over 72 h in the presence or absence of 1 μM rVλ6Wil fibrils by cell counting using fluorescence microscopy. The number of nuclei stained with Hoescht 33342 was evaluated in a minimum of two low power (5x objective magnification) fields of view.

A cell viability assay was performed by using fluorescence microscopy with CMFDA. Briefly, cultured AC10 cells were grown for 4 days on 15 mm coverslips in 12-well tissue culture plates and exposed to 1μM rVλ6Wil fibrils for 24, 48, and 72 h in serum free DMEM/F12. On day 4 of cell growth, culture medium was removed and the coverslips treated with 1 mL of pre-warmed cell culture medium containing 5 μM CMFDA for 15 min in a dark cell incubator. The cells were washed once with warm PBS, and culture medium replaced for a further 15 min in the dark before being washed a final time in PBS and fixed using 4% paraformaldehyde. Samples were stored at 4°C in PBS for less than 24 h before being counterstained with Hoescht 33342 and examined microscopically with 20x objective magnification using a Leica DMR 500 microscope with epifluorescent illumination. Digital images were captured using a cooled CCD camera (RT-slider, SPOT Imaging Solutions, Sterling Heights, MI) using the monochrome chip. For each sample, four fields of view containing at least 150 cells per field were analyzed. The number of dead cells—those that fail to retain the green fluorescence dye—were counted and expressed as a % of total cells in the field of view.

### Measurement of cellular ATP

ATP levels were measured in rVλ6 monomer or fibril-treated cells as follows. Cells were seeded at 2 x 10^4^/cm^2^ in a tissue culture-treated opaque-walled black 96-well plate (BD Biosciences, Franklin Lakes, NJ) and grown for 24 h before changing to serum-free DMEM:F12 and treating with rV_L_λ6Wil proteins. At the end of the exposure period, ATP was detected using the Promega CellTiter-Glo Luminescent Cell Viability Assay. The microplates were equilibrated to room temperature and 100 μL of the CellTiter-Glo reagent was added to each well; after 2 min of gentle shaking followed by a 10 min incubation at room temperature, luminescence intensity was measured using a Wallac Victor 2 Multilabel plate reader (Perkin Elmer, Waltham, MA).

### Kinetic analysis of MTT assay following exposure to rVλ6Wil

Cardiomyocytes were seeded in 24-well plates either at high density (HD), 2 x 10^4^/cm^2^, or low density (LD), 1 x 10^4^/cm^2^, in growth medium. After 24 h, the growth medium was replaced with serum-free DMEM:F12 and rV_L_λ6 Wil fibrils were added to a final concentration of 1 μM at 1, 2, 12, 18, or 24 h prior to performing the MTT reduction analysis. Just prior to the addition of MTT, the cells were washed once with serum-free medium to remove the rVλ6Wil fibrils and fresh serum-free medium was added to the wells. After 4 h of MTT reduction at 37°C, the activity was determined as described above.

### Fractionation of rVλ6Wil fibril preparations

A suspension of freshly prepared rV_L_λ6Wil fibrils was centrifuged at 435,000 x g for 15 min (Optima TLX with TLA 100 rotor; Beckman, Pasadena, CA) or 16,000 x g for 15 min and the supernatants isolated for evaluation in the MTT-reduction assay in a 1x (1 μM) or 10x (10 μM) volume-equivalent to the unfractionated stock. In addition, an rVλ6Wil fibril suspension was passed through a 100,000 MWCO Amicon Ultra filtration unit (EMD Millipore, Billerica, MA) by centrifugation at 4,000 x g for 15 min and the filtrate was used in a volume corresponding to 1 μM of original concentration assayed in the cell MTT reduction assay.

Reverse phase high performance liquid chromatography (HPLC) was performed using a C3 solid matrix (Zorbax SB-C3; Agilent Technologies, Santa Clara, CA) with an acidified (0.05% trifluoroacetic acid) acetonitrile mobile phase of increasing gradient from 1–51% (2% ACN per min). Protein elution was detected by monitoring the absorbance at 215 nm.

### Immunofluorescence Microscopy of Wil Fibril Binding

AC10 cells were plated in growth medium at 20–30% confluency on 15 mm glass coverslips in 12-well tissue culture plates. After 24 h the cells were washed once, the medium replaced with serum-free DMEM:F12, and fluorescent rVλ6Wil fibrils at 1 or 2 μM were added. At the designated time points, the cells were washed 3x with PBS and fixed for 20–30 min by addition of 4% paraformaldehyde (BD Cytofix). After fixation, cells were further washed and stored in PBS until analyzed. For visualization of the cytoskeletal F-actin, the cells were permeabilized by addition of 0.2% Triton X-100, followed by PBS/1% BSA (PBSA) for 5–10 min, and incubated with Alex Fluor 647-labeled phalloidin in PBSA (10 μL per coverslip) for 1 h. Nuclear staining was then performed by incubating the coverslips in PBS containing 5 μg/mL Hoescht 33342. After 2 further washes in PBS, the coverslips were mounted on glass slides using a fluorescent mounting medium (Dako, Carpinteria, CA) and observed using a Leica DMR 500 microscope with epifluorescent illumination. Digital images were captured using a cooled CCD camera (RT-slider, SPOT Imaging Solutions, Sterling Heights, MI) using the monochrome chip.

Quantification of fluorescent rVλ6Wil fibril area and cell nuclei was performed using the Image Pro Premier image analysis software (ver. 9.0; Media Cybernetics, Rockville MD). Three fields of view, captured at 10x objective magnification, were analyzed for each time point (1, 2, 6, and 24 h post fibril addition). To ensure accurate representation of the culture, there was a minimum of 50 cell nuclei per field of view.

Confocal microscopy was performed on cells, treated and untreated, grown on glass coverslips and stained as described above. The cells were imaged using a LSM 710 confocal laser scanning microscope with a Plan-Aprochromat x63/1.40 oil immersion objective (Carl Zeiss Microimaging, Thornwood, NY, USA). Optical sections were collected at 0.1-μm z-step distance and processed using Zen 2010 software (Carl Zeiss Microimaging).

### Cardiac amyloid tissue staining

Cardiac tissue obtained at autopsy, with informed consent, from a patient with AL cardiomyopathy was fixed in 4% buffered formalin and 6-μm sections cut and placed on glass slides. Detection of amyloid was achieved by staining with an alkaline Congo red solution (0.8% w/v Congo red, 0.2% w/v KOH, 80% ethanol) for 1 h at room temperature followed by counterstain with Mayer’s hematoxylin for 2 min. Sections were viewed under cross polarized illumination using a Leica DMR 500 microscope (40x objective). Amyloid was stained by using a biotinylated amyloid-reactive peptide as previously described [36]. The presence of amyloid was visualized by the addition of diaminobenzidene and evidenced as a brown coloration. Images were acquired using brightfield transmitted illumination (40x objective).

### Detection of ROS in cardiomyocytes

Production of intracellular reactive oxygen species was measured using DCFH-DA, a redox-sensitive fluorophore [37]. After exposing AC10 cardiomyocytes to 1 μM rVλ6Wil fibrils for either 2 or 20 h in 12 well tissue culture-treated plates, cells were washed and incubated with 50 μM DCFH-DA for 45 min at 37°C. The dye solution was replaced with HBSS and cells were incubated for an additional 30 min at 37°C. For a positive control, DCFH-DA-treated AC10 cells in HBSS were exposed to serial dilutions of 30% H_2_O_2_ for 30 minutes at 37°C. For fluorescence quantitation, the HBSS was replaced with 200 μL per well 90% DMSO/10% PBS and plates were shaken in the dark for 10 min [38]. The contents of each of the 12 wells was transferred to a separate well in an opaque-walled black 96-well microtiter plate and the fluorescence at 535 nm emission (excitation = 488 nm) was measured using a Wallac Victor^2^ Multilabel plate reader (Perkin Elmer).

### Measurement of oxygen consumption rate

The effects of fibrils or monomers on oxidative metabolism of AC10 cells, as defined by the oxygen consumption rate (OCR), were examined using a Seahorse XF24 Analyzer (Seahorse Biosciences; North Billerica, MA). Briefly, AC10 cells were plated one day prior to assay. On day of assay, cell culture medium was exchanged for serum-free medium 2 h prior to addition of fibrils or monomers diluted in media to achieve a final concentration of 1 μM. A PBS-only, no fibrils, or monomer protein, injection was used as a control. OCR was monitored throughout the duration of the experiment. The data are expressed as the average (n = 5 wells/condition) %OCR from baseline or before injection of fibrils or monomer over a 2 h period of observation after the addition of test agent. The experiment was repeated twice.

### Statistical analyses

Statistical tests of significance (t-test and ANOVA) and column statistics were calculated using Prism 6 (ver. 6.05; GraphPad Software, Inc). Oxygen consumption rate data were analyzed by first calculating the Area Under the Curve for each condition and significant differences were determined by using an ANOVA followed by a Post-hoc Tukey’s test. **p-value < 0.01; *p-value < 0.05.

## Results

**Fig 2:**
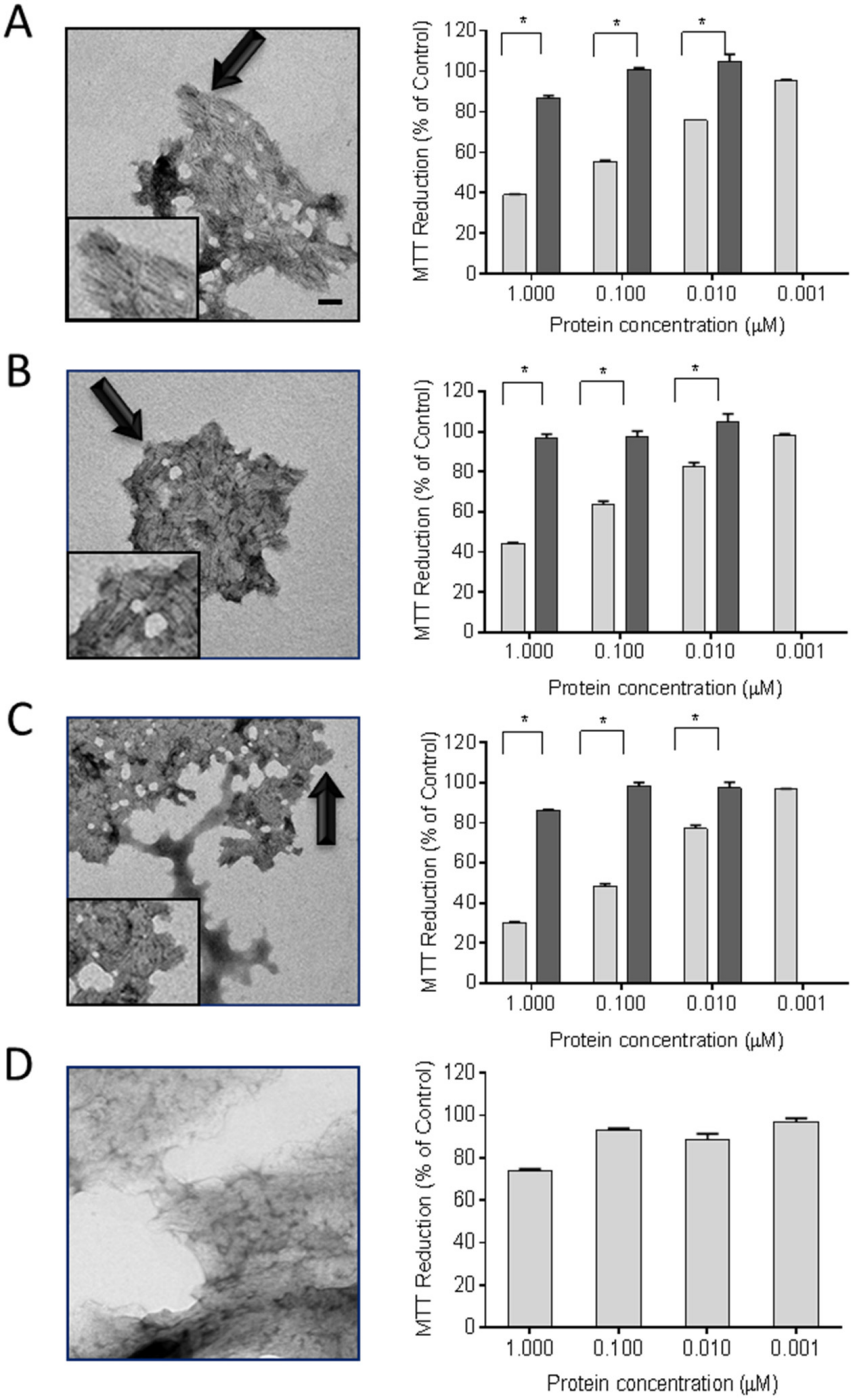
rVλ6 amyloid fibrils cause metabolic dysfunction in cultured cardiomyocytes. Synthetic fibrils composed of rVλ6Wil (A), Jto (B), JtoR68S (C), and non-amyloid fibrils elastin (D) were shown to be fibrillar by using electron microscopy (left panels; bar = 100 nm; arrows indicate the region of fibrils shown inset). MTT reduction was measured in cultured AC10 human cardiomyocytes in the presence of fibrils (light) and monomeric proteins (dark) from 1 nM– 1 μM. *p<0.05, for an unpaired t-test (right panel, n = 3). EM images were modified equivalently by increasing contrast and applying a shadowing effect, using Image J.

The metabolic activity of cultured AC10 cardiomyocytes was measured in the presence of two patient-derived rVλ6 (Wil from an AL amyloidosis patient and Jto from a multiple myeloma patient) as well as one point mutant with enhanced fibrillogenic potential (JtoR68S), either in fibrillar or monomeric forms (Fig 2). Purified monomer protein samples were used fresh or thawed from frozen storage. Monomeric preparations of rVλ6 proteins were ThT-negative (assumed to be non-fibrillar) and shown to be >95% monomer by RP-HPLC analyses (data not shown). Monomer or fibrillar (f) rVλ6Wil, Jto, or JtoR68S were added to AC10 cardiomyocytes plated 24 h earlier. The concentrations of fibrils were based on the amount of monomer incorporated into fibrils, which was shown by RP-HPLC to be >95%. The ultrastructure of the ThT-positive rVλ6 aggregates following 72 h of shaking was shown, by using transmission electron microscopy, to comprise short linear fibrils that had associated longitudinally to form compact sheets (Fig 2A–2C). Non-amyloid, i.e., ThT-negative, fibrils composed of elastin served as a control (Fig 2D). Incubation of rVλ6 fibrils with the cardiomyocytes for 24 h caused a dose-dependent decrease in the reduction of MTT (Fig 2A–2C). The most dramatic reduction in MTT oxidation was seen in the 1 μM fWil and fJtoR68S samples with a 61% and 70% reduction, relative to the PBS control, respectively. For all fibrillar preparations there was a significant decrease in MTT reduction (*p* < 0.05) at 0.01 μM protein (Fig 2). In contrast, treatment of the cardiomyocytes with non-fibrillar rVλ6 proteins resulted in a maximal 15% decrease in MTT reduction at 1 μM protein, relative to the control; however, this was significant (*p* < 0.005) for rVλ6Wil and rVλ6JtoR68S relative to the 0.01 μM monomer treatment group (Fig 2A–2C). The monomeric rVλ6Jto sample caused only a ~5% reduction in MTT signal, which was not significant (Fig 2B). Non-amyloid elastin fibrils required 1 μM protein to cause a significant (*p* < 0.05) 25% decrease in MMT reduction (Fig 2D).

**Fig 3:**
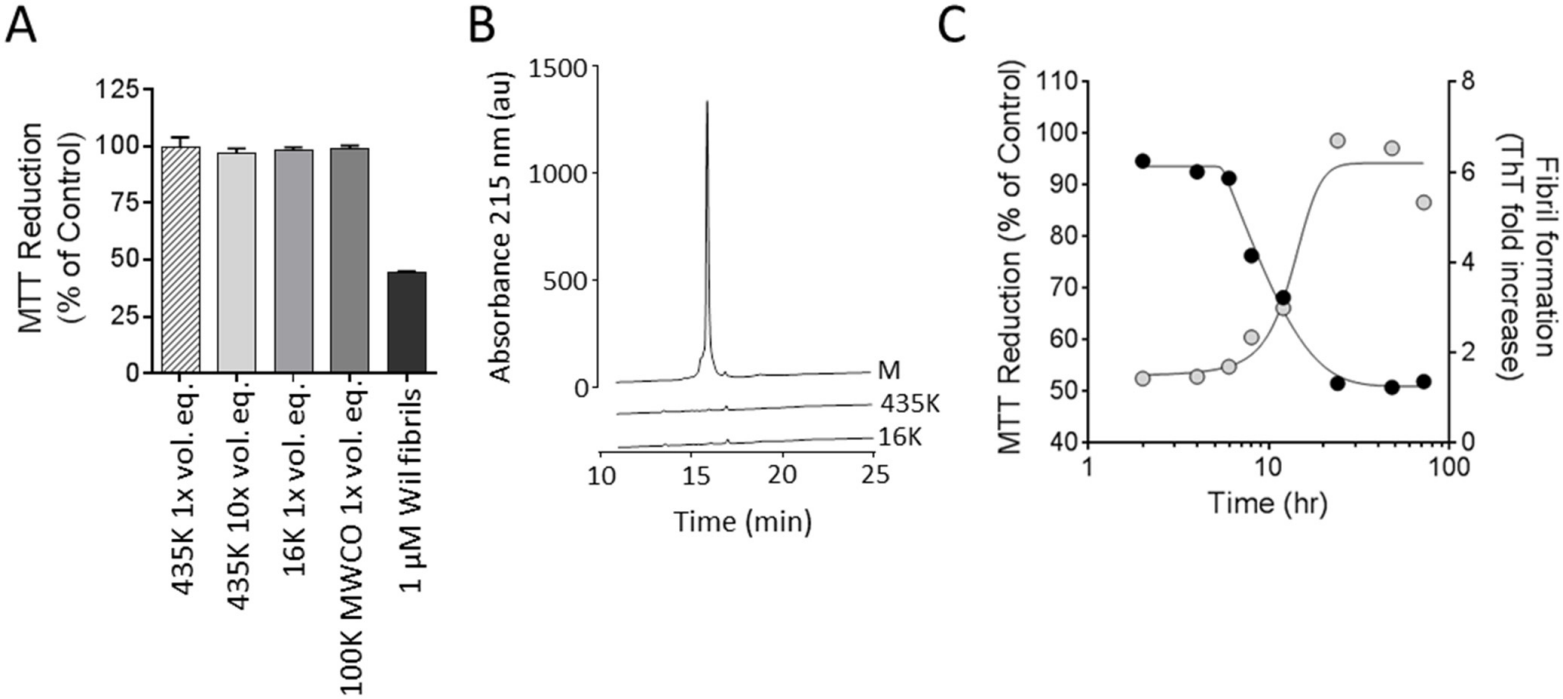
Metabolic dysfunction of cardiomyocytes is dependent on the presence of rVλ6Wil fibrils. (A) Supernatants from a suspension of rVλ6Wil fibrils generated by centrifugation of a fibril suspension at either 435,000 x g or 16,000 x g do not decrease MTT reduction by cardiomyocytes (supernatants were used at a 1x or 10x volume equivalent [vol. eq] to the unfractionated fibril suspension). Filtrate from a suspension of rVλ6Wil fibrils passed through a 100,000 (100K) MWCO filter did not contain material that affected MTT reduction (n = 3, per assay). (B) HPLC analysis of supernatant samples from the 435,000 x g (435K) and 16, 000 x g (16K) centrifugation as compared to the same volume of starting rVλ6Wil monomer (M) material. (C) Over the course of rVλ6Wil fibrillogenesis, only samples of the reaction mixture containing ThT-positive material significantly decreased MTT reduction when added for 24 h to AC10 cardiomyocytes. Gray circles, thioflavin T fluorescence (excitation = 450 nm; emission = 490 nm); black circles, MTT reduction (n = 3 sample per time point).

Based on our observation that the fibrillar form of rVλ6 proteins was more potent at causing metabolic dysfunction in cultured cardiomyocytes as compared to the homologous monomer protein, we sought to characterize the molecular species in the rVλ6Wil sample responsible for this effect in greater detail. Centrifugation at 435,000 (435K) or 16,000 (16K) x g or filtration through a 100,000 (100K) MW cut-off filter were used to separate larger fibrillar aggregates from soluble monomer (and possibly oligomeric species) present in the preparation. The supernatants from these treatments, lacking precipitable fibrillar material, were evaluated in the MTT reduction assay and the results compared to those using an unfractionated Wil fibril sample (Fig 3A). In short, all treatments resulted in fibril-free supernatants (no detectable ThT fluorescence) that caused no significant decrease in the MTT reduction assay even when tested at 10 x the volume used for the fibril treatment (Fig 3A). The supernatant samples from the 435,000 x g and 16,000 x g centrifugation steps, as well as the rVλ6Wil starting material, were analyzed by using reverse phase HPLC (Fig 3B). The monomer rVλ6Wil (M; 1 mg/mL) eluted at ~ 16 min. Based on area under the curve measurements, only ~ 1% of the starting material was present in supernatants generated by centrifugation at 435,000 (435K) or 16,000 (16K) x g (Fig 3B).

To investigate at what point the “cardiotoxic” species appear during the process of fibril formation, a preparation of rVλ6Wil monomer at 1 mg/mL (83 μM) in PBS was incubated at 37°C while shaking to generate fibrils. Samples were taken from the reaction mixture at 1, 2, 4, 6, 8, 12, 24, 48 and 72 h and added at 1 μM final concentration to AC10 cardiomyocytes for assay of MTT reduction (Fig 3C). Under these conditions, the presence of ThT-positive fibrils occurred between 8 and 24 h of incubation, which was consistent with the time of decrease in MTT reduction (Fig 3C). During the lag phase of fibril formation (0–8 h), there was no evidence of cardiotoxic species as indicated by the absence of change in the MTT reduction assay, relative to PBS-treated control cells (Fig 3C).

**Fig 4:**
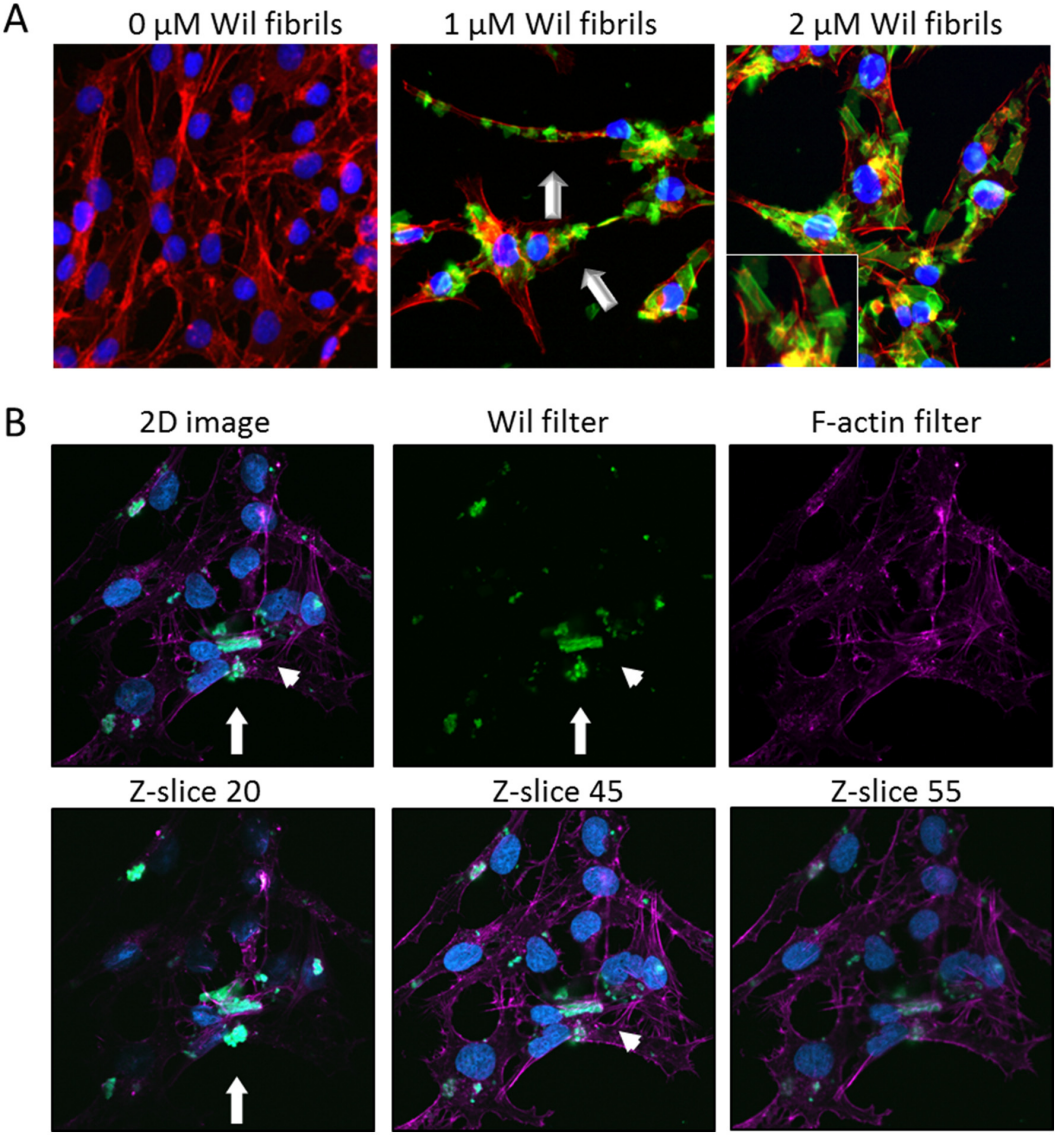
rVλ6Wil fibrils bind cultured cardiomyocytes. (A) Fluorescent synthetic fibrils composed of rVλ6Wil (Alexa Fluor-488, green; arrow) bound specifically to cultured AC10 cardiomyocytes (blue, nuclei; red, f-actin). (B) Confocal micrographs of cultured cardiomyocytes (blue, nuclei; purple, f-actin) after incubation with 1 μM fluorescent rVλ6Wil fibrils (green). Optical sections at the surface (Z-slice 20), center (Z-slice 45), and bottom (Z-slice 55) demonstrated the presence of fibrils on the cell surface (arrow) and rare intracellular material (arrowhead). All images were enhanced equivalently by increasing the brightness.

The interaction of rVλ6Wil fibrils with cultured AC10 cardiomyocytes was examined using fluorescent, Alexa Fluor488-labeled fibrils (green; Fig 4). Fibrils formed in the presence of 10% fluorescent rVλ6Wil monomer were added to cells grown on glass coverslips at 1 or 2 μM final concentration for 24 h. In 2D microscopy images (Fig 4A, inset), the fibrils appeared as “sheets” or large aggregates, consistent with the pattern of macroscopic aggregation seen in the electron micrographs (Fig 4A, inset). The cells appeared coated with fibril aggregates in a specific but heterogeneous fashion (Fig 4). Notably there was little evidence of non-specific adsorption of the fibril aggregates to the coverslip (Fig 4A). The F-actin distribution visualized by the Alexa Fluor 594-labeled phalloidin (red) appeared to be intact and unaffected by the addition of the rVλ6Wil fibrils, when compared to staining patterns of the untreated cells (data not shown). Using the same fluorescence assay, there was no evidence of rVλ6Wil monomer binding to the AC10 cells under identical experimental conditions (data not shown).

Three-color confocal microscopy was used to determine whether the amyloid fibrils were extracellular or had been internalized during the 24 h incubation period (Fig 4B). Analysis of these images indicated that the large aggregates were predominantly extracellular and associated intimately with the cell surface (Fig 4B, arrow); however, in a few cells, there was evidence of internalization of small fluorescent aggregated material (Fig 4B arrowhead; Z-slice 45).

**Fig 5:**
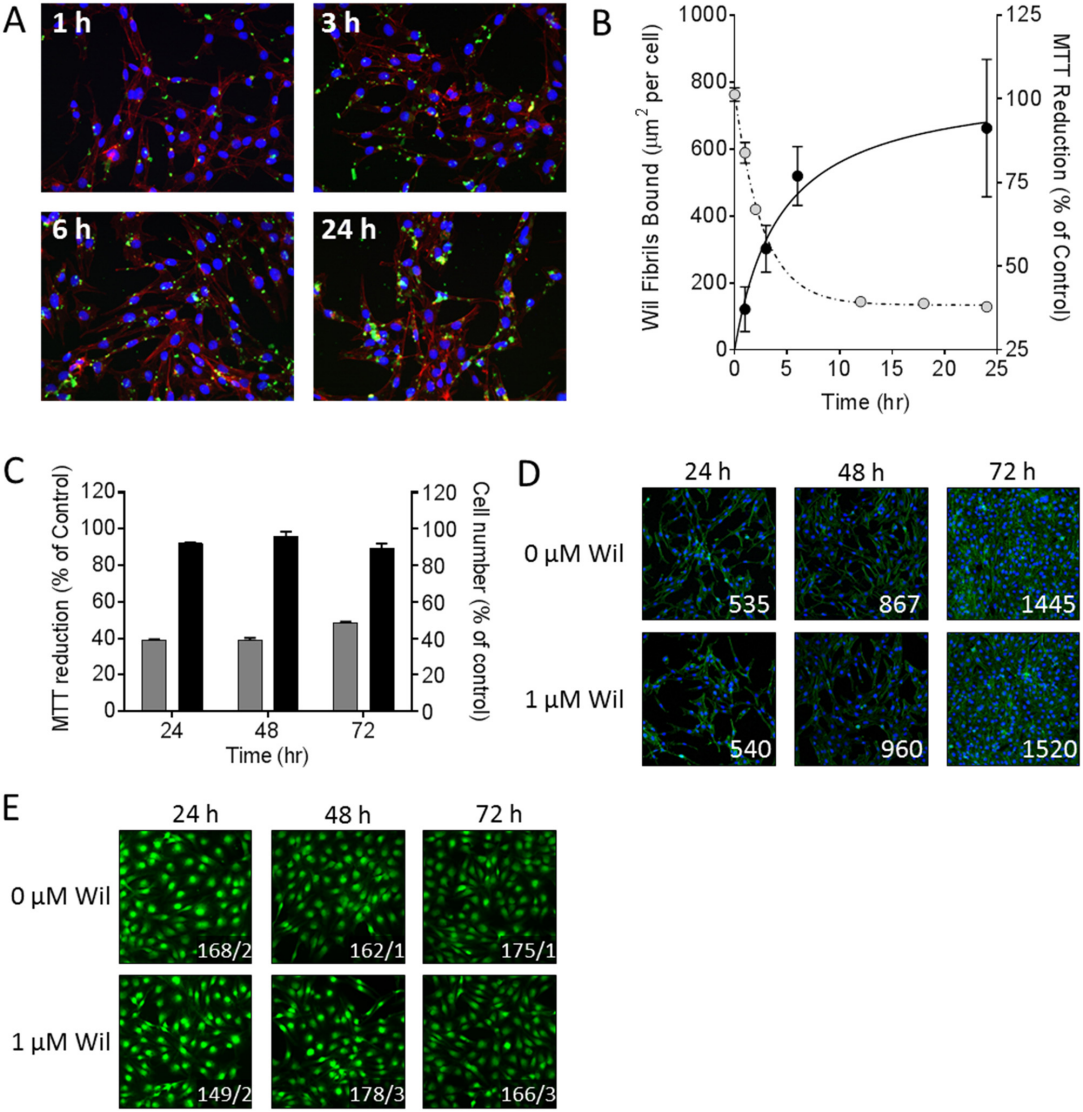
rVλ6Wil fibrils cause a decrease in cardiomyocytes MTT reduction without inducing cell death. (A) Fluorescence microscopy demonstrating the increase in binding of Alexa Fluor-488 labeled rVλ6Wil fibrils (1 μM) over 24 h of incubation (blue, nuclei; red, f-actin). (B) Quantitation of rVλ6Wil fibril binding to cardiomyocytes (μm^2^/cell; closed circles) correlated inversely, over 24 h, with MTT reduction (open circles). (C) Cell number, quantified using crystal violet (black) did not decrease over 72 h of incubation with 1 μM rVλ6Wil fibrils despite a decrease in MMT reduction (grey; n = 3 samples per time point). (D) Fluorescence micrographs of AC10 cardiomyocytes incubated with or without non-fluorescent 1 μM rVλ6Wil fibrils (blue, nuclei; green, f-actin; numbers represent cell count in 10x objective field of view). (E) Cell viability of AC10 grown in culture for 4 days in the presence or absence of 1 μM rVλ6Wil fibrils for 24, 48, or 72 h, was assessed by CMFDA fluorescence (original objective, 20x. Images were cropped and digitally magnified 4x. Numbers represent the mean live/dead cells, *n = 4* independent fields of view).

The kinetics of rVλ6Wil fibril binding to cardiomyocytes and MTT reduction was studied by quantifying, from 2D fluorescent microscope images, the cell-bound fluorescent amyloid (expressed as μm^2^ per cell by counting nuclei in the field of view) and comparing this with MTT reduction in the same time interval (Fig 5). There was an increase in the binding of rVλ6Wil fibrils over the 24 h period of analysis (Fig 5A) and a concomitant decrease in cardiomyocyte metabolic activity, as measured by MTT reduction (Fig 5B). The kinetics of fibril binding and metabolic dysfunction were compared by estimating the *T*_1/2_ values for both effects by using non-linear curve fitting, yielding values of 2.5 and 1.9 h, respectively (Fig 5B). Cell number in the presence or absence of 1 μM rVλ6Wil fibrils was assessed by measuring crystal violet (Fig 5C) or direct cell counting of adherent cells grown on glass coverslips (Fig 5D). In both analyses, there was no evidence of a significant difference in the cell number associated with incubation with 1 μM fibrils over 72 h as compared to control (PBS-treated) cells. Cell viability of AC10 cells grown for 4 d in the presence of 1 μM rVλ6Wil fibrils for 24, 48, or 72 h of incubation was assessed by using CMFDA; Fig 5E). At 24, 48, and 72 h of fibril exposure, the number of dead cells (% total) in the presence of fibrils was 0.7 ± 0.9, 1.5 ± 0.8, and 1.5 ± 0.6, respectively.

**Fig 6:**
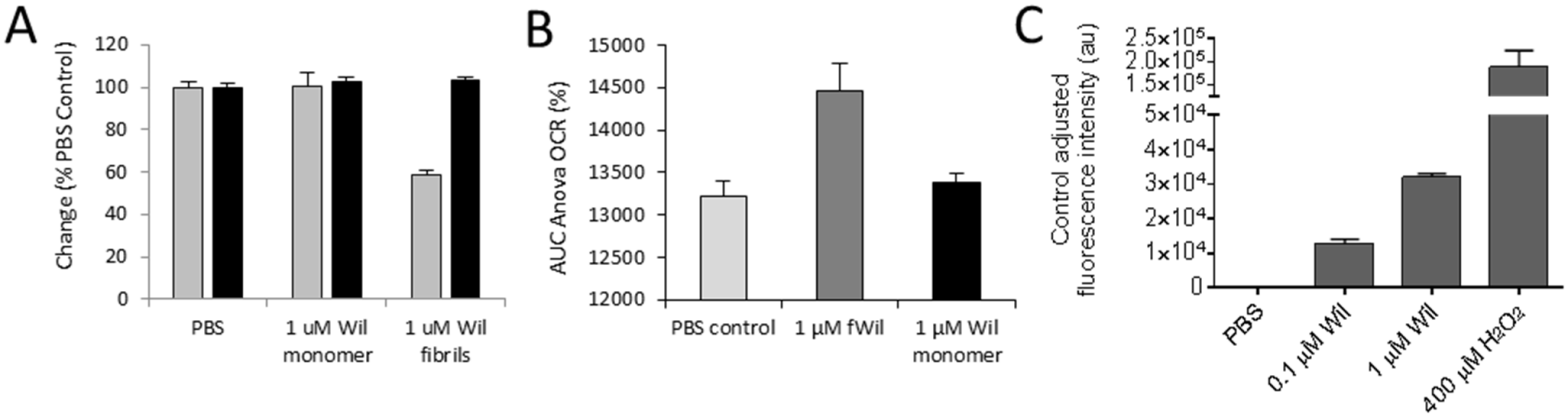
rVλ6Wil fibrils cause oxidative stress in cardiomyocytes without a decrease in cellular ATP. (A) After a 2 h incubation with rVλ6Wil fibrils or monomer, cellular ATP levels, as detected by luminescence (black bars), were not significantly reduced relative to the PBS-treated control. A decrease in MTT reduction (gray bars) was observed in the presence of fibrils (n = 3). (B) Oxygen consumption rate was significantly higher in AC10 cells (*p* < 0.05) treated with 1μM rVλ6Wil fibrils as compared to treatment with PBS or rVλ6Wil monomer protein (n = 6 wells). (C) Cardiomyocytes labeled with DCFH-DA exhibited a dose-dependent increase in ROS production after 24 h of exposure to rVλ6 Wil fibrils (n = 3).

To complement our findings of fibril-mediated metabolic dysfunction in cultured cardiomyocytes by measuring MTT reduction, we assessed other aspects of cell function and dysfunction in response to the fibrils. Notably, ATP levels, oxygen consumption, and the generation of reactive oxygen species were measured in response to fibril exposure (Fig 6). The reduction of cellular ATP was measured after a 2 h or 24 h incubation of cells with either 1 μM rVλ6Wil monomer or fibril (data for 2 h incubation only shown in Fig 6A). There was no decrease in the level of cellular ATP under any conditions tested. In parallel, MTT reduction was measured over the same time interval and, consistent with previous data, the presence of rVλ6Wil fibrils resulted in a ~50% decrease in MTT reduction (Fig 6A). The oxygen consumption rate (OCR) of proliferating cardiomyocytes, an indicator of mitochondrial respiration, was measured by using an XF24 Analyzer (Fig 6B).

Treatment of AC10 cardiomyocytes with 1 μM fibrils, but not the monomer or PBS control, resulted in a significant (*p* < 0.05) increase in the OCR (displayed as the % change in the OCR) over a 2 h measurement period. Finally, we assessed whether reactive oxygen species were generated in response to the binding of rVλ6Wil fibrils (Fig 6C). After a 24 h fibril incubation, there was a dose-dependent increase in ROS in the presence of fibrils as compared to cells treated with PBS alone. The assay was validated by incubating the cells in 400 μM H_2_O_2_, which resulted in extensive ROS production (Fig 6C). These data indicate, that in addition to altered cellular metabolic activity manifest in a decrease in MTT reduction, amyloid fibrils also increased cellular oxygen consumption rate, which may have contributed to the generation of intracellular ROS.

## Discussion

Light chain cardiomyopathy is a common feature of patients with AL. The incessant accumulation of infiltrative amyloid in the myocardium causes a restrictive cardiomyopathy with reduced ventricular contractility and impaired relaxation [10, 13, 14]. In addition to extracellular amyloid deposits, cardiomyocytes are also exposed to LC in the circulation and extra-vascular fluid, which may be cytotoxic and also contribute to cardiac dysfunction. The potential cardiotoxic effect of amyloid fibrils themselves on cardiomyocyte metabolic integrity has largely been neglected and represents another source of potential organ damage in these patients.

To investigate this, we focused on measurement of MTT reduction, a measure of cytosolic and intracellular membrane NAD(P)H oxidoreductase activity (when cell number remains constant), as an indicator of metabolic dysfunction in a cultured human cardiomyocyte cell line. The λ6 LC variable region proteins used in our study were made by recombinant methods using amino acid sequences obtained from patients Wil and Jto who presented with primarily renal amyloidosis and multiple myeloma, respectively. The rVλ domains, as well as the point mutant rVλ6JtoR68S [30, 33] are highly fibrillogenic under physiological conditions with >95% conversion of monomer to fibril, and were thus a perfect model system to assess the cardiotoxic effects of fibrils. We demonstrated that at concentrations as low as 10 nM rVλ6 fibrils there was a significant decrease in MTT reduction relative to the equivalent concentration of monomeric rVλ6 protein and the vehicle control. Significant metabolic dysfunction was not induced by the monomeric rVλ6 protein until 1μM (~0.12 mg/mL) was reached, indicating that the fibrils were 2 orders of magnitude more toxic in this assay. It should be noted that, in other studies of LC toxicity on cardiomyocytes, a detrimental toxic effect was detected only at these higher concentrations [23, 24].

Monomeric rVκ1 proteins from patients with AL amyloidosis AL-09 and AL-12, as well as the germline O18/O8 variable domain protein have been shown previously to decrease MTT reduction in cultured murine HL-1 cardiomyocytes. These proteins were shown to be internalized and to cause apoptosis in the absence of fibril formation [25, 39]; however, the concentrations tested in these studies were 10-fold greater than used for our experiments on monomer proteins. In addition, there was a correlation between rVκ1 domain stability, enhanced fibrillogenic potential and the induction of metabolic dysfunction and apoptosis [39]. Interestingly, the rVλ6Wil and rVλ6JtoR68S monomeric proteins, which are thermodynamically less stable as compared to Jto (Δ*G*_H2O_ = 4.3, 3.2, and 2.2 Kcal/mol for Jto, JtoR68S, and Wil, respectively) [30] were significantly more toxic than Jto when tested at 1 μM. These data support supposition that the thermodynamic instability of rVκ and rVλ correlates with induction of cardiomyocyte dysfunction.

The toxicity of rVλ6Wil fibrils (0.1 μM) in our system was equivalent to that observed for the non-fibrillar form of rVκAL-09 (~12 μM; [25]). In addition, the rVκAL-09 was rapidly internalized by cultured HL-1 and sequestered in the lysosomal compartment [39], suggesting that this may be required for induction of cytotoxicity, which stands in contrast to fibrillar forms that likely exert their effect extracellularly. Using fluorescence microscopy, we demonstrated the accumulation of rVλ6Wil fibrils on the surface of cultured AC10 cells with no evidence of extensive internalization (as evidenced by confocal microscopy). These data suggest that the metabolic dysfunction is mediated via an interaction with cell surface components, possibly proteoglycans, such as perlecan, that are known to be intimately associated with amyloid deposits *in vivo* [40].

The decrease in MTT reduction seen in our system suggests that the rVλ6 fibrils induced metabolic dysfunction via inhibition of NAD(P)H oxidoreductase activity. This observation contrasts in several ways with other studies of LC cardiotoxicity. For example, Migrino *et al*. using human adipose and coronary arterioles demonstrated a reduction in dilatory response to chemical stimuli after exposure to amyloidogenic LC proteins but not heat denatured LC or those from patients with multiple myeloma [41]. The underlying mechanism was shown to be apoptosis resulting from oxidative stress in the arteriole endothelia, which was reversible upon treatment with superoxide dismutase and tetrahydrobiopterin antioxidants [41].

It is a common theme in published studies that LC derived from myeloma patients and indeed those from AL patients, without severe AL cardiomyopathy, are generally less cytotoxic to cardiomyocytes, suggesting that only a discrete subset of amyloidogenic LC cause metabolic dysfunction in cardiomyocytes. Liao *et al*. demonstrated using isolated mouse hearts, that LC from patients with severe cardiac AL (severe hypertrophy with NYHA class III or IV heart failure) used at micromolar concentrations, caused a significant increase in left ventricular end diastolic pressure, but LC from AL patients with mild (NYHA I and II) or no cardiac impairment did not elicit this effect [26]. Furthermore, using isolated rat cardiomyocytes, LC from cardiac AL patients elicited impaired contractility and calcium transport, which reversed by inclusion of a superoxide dismutase mimetic. Notably, there was no evidence of LC oligomerization or fibrillogenesis in this system, implying that the free LC monomer, dimer or misfolded population thereof was the toxic species [23]. The underlying mechanism was shown to involve oxidative stress via activation of the non-canonical p38α MAPK pathway and upregulation of stanniocalcin1 [24, 27]. Similar MAPK activation and cytotoxicity had been shown to be associated with neurodegeneration in patients with amyloidogenesis in patients with Alzheimer’s disease [42].

To investigate further whether an oligomeric (non-fibrillar) component of the rVλ6Wil protein was responsible for the observed metabolic dysfunction of the AC10 cells, we performed a series of “fractionation” steps to isolate fibrils from the suspension. In each case, the resulting supernatant caused no decrease in MTT reduction Indeed, even with only mild (16,000 x g) centrifugation, there was no evidence of significant rVλ6Wil in the resulting supernatant (Fig 3B), further indicating that ~ 99% of rVλ6Wil protein formed fibrils and that these fibrils, and not monomer, constitute the toxic entity. By sampling a fibrillogenesis reaction, we were able to demonstrate that metabolic dysfunction was only achieved when the reaction mixture contained ThT-positive material, indicative of the presence of amyloid fibrils (Fig 3C).

Apoptosis is a common endpoint in previous studies of LC toxicity seen in both arteriole endothelia and cardiomyocytes [23, 25, 41, 43]. In our studies, there was no evidence of cell death or reduction of cell growth rate in the presence of rVλ6Wil fibrils over 72 h of incubation even when metabolic dysfunction was manifest (Fig 5).

We have demonstrated that incubation of rVλ6Wil fibrils for as little as 2 h resulted in an increase in oxygen consumption rate relative to monomer or vehicle controls. Since there was no decrease in cellular ATP over the same time period, this suggests that the fibrils metabolically uncouple respiration from ATP production [44]. Similar metabolic uncoupling has been observed by treating cells with 2, 4 –dinitrophenol (2,4 –DNP), which led to elevated OCR, with a concomitant decrease or no effect on ATP levels[44]. However, in contrast to 2, 4-DNP, which has been shown to reduce ROS levels [45], treatment of AC10 cardiomyocytes with fibrils increased ROS levels. Interestingly, it has been suggested that mitochondrial uncoupling of respiration and ATP production may act as a protective mechanism to prevent cellular ROS formation [46, 47]. Our data indicate that the fibrils did not increase OCR immediately, but rather after ~100 min of exposure. The monomer, which had no effect on ROS levels in AC10 cardiomyocytes, also failed to alter OCR. Thus, increasing OCR with no change in ATP levels may represent a protective mechanism adopted by the cardiomyocytes in response to the fibril-mediated elevation in cellular ROS. The increase in oxygen consumption and the subsequent decrease in MTT reduction are consistent with MTT reduction occurring in the cytosolic compartment [48]. The activity of NADPH-dependent oxidase enzymes is governed through increased NADPH in conjunction with oxygen. However, an increased OCR would not necessarily result in elevated MTT reduction, especially if NADPH levels are low. The precise mechanism by which fibrils alter cellular respiration and whether this effect is specific to cardiomyocytes remains enigmatic.

Amyloidosis is a complex disease process with numerous pathological sequelae. Without doubt, the progressive accumulation of amyloid in the heart and other organs contributes to architectural damage and organ dysfunction. The potential for free LC cardiotoxicity in patients, or indeed toxicity to other organs and tissues [49, 50], and the presence of damaging oligomeric LC intermediates acting locally or systemically is presently poorly understood. Our results present the possibility that amyloid fibrils, acting through cell-surface proteins or proteoglycans, can elicit oxidative stress and metabolic dysfunction in the absence of cell death/apoptosis, which may contribute to mortality and morbidity. Thus, in addition to striving for hematologic response using well-established chemotherapy and other novel anti-plasma cell therapies, it may be equally important to facilitate removal of tissue amyloid as a means of restoring organ function thereby securing long term survival and remission in patients. To this end, several anti-amyloid immunotherapeutics, designed to induce cell-mediated clearance of amyloid, are being developed and evaluated in clinical trials (clinicaltrials.gov # NCT01707264 [mAb NEOD001; [51]] and NCT02245867 [mAb 11-1F4; [52]]). When incorporated into the standard of care for AL patients, these novel reagents may improve clinical outcomes and survival.

## Abbreviations

rVλ6: recombinant λ6 immunoglobulin variable domain
LC: immunoglobulin light chain
MTT: 3-(4,5-dimethylthiazol-2-yl)-2,5-diphenyltetrazolium bromide
DMEM:F12: Dulbecco’s modified eagles medium: F12
DCFH-DA: 2'-7'-dichlorodihydrofluorescein diacetate
DMSO: dimethylsulfoxide
EDTA: ethylenediaminetetraacetic acid
ROS: reactive oxygen species
ThT: Thioflavin T
CMFDA: 5-chloromethylfluorescein diacetate
